# The impact of preformed donor‐specific antibodies in living donor liver transplantation according to graft volume

**DOI:** 10.1002/iid3.586

**Published:** 2022-01-22

**Authors:** Ryoichi Goto, Makoto Ito, Norio Kawamura, Masaaki Watanabe, Yoshikazu Ganchiku, Toshiya Kamiyama, Tsuyoshi Shimamura, Akinobu Taketomi

**Affiliations:** ^1^ Department of Gastroenterological Surgery I Hokkaido University Graduate School of Medicine Sapporo Japan; ^2^ Division of Laboratory and Transfusion Medicine Hokkaido University Hospital Sapporo Japan; ^3^ Department of Transplant Surgery Hokkaido University Graduate School of Medicine Sapporo Japan; ^4^ Division of Organ Transplantation Hokkaido University Hospital Sapporo Japan

**Keywords:** GV/SV ratio, left hepatic lobe, lymphocyte crossmatch test, pre‐transplantation, small for size graft

## Abstract

**Introduction:**

The roles of preformed anti‐HLA donor‐specific antibodies (DSAs) in liver transplantation remain controversial. We evaluated the impact of preformed DSAs in living donor liver transplantation.

**Methods:**

Adults who underwent living donor liver transplantation (*n* = 175) in our institute were included in this study. Lymphocyte cytotoxicity test (LCT), flow cytometric crossmatch (FCXM), and single‐antigen bead assays were performed.

**Results:**

Among adult living donor liver transplantation recipients, 27 (16.5%) and 14 (8.5%) had pretransplant FCXM‐positive findings and LCT‐positive findings, respectively. FCXM‐positive patients displayed a significantly worse 5‐year graft survival rate (77.3%; vs. DSA‐negative, 91.6%). Six of 14 LCT‐positive patients exhibited graft loss shortly after transplantation (5‐year survival rate: 57.1%). All LCT‐positive patients with graft loss underwent left lobe living donor liver transplantation. Significantly lower ratio of graft volume relative to standard liver volume (32.9 ± 5.7%) and smaller graft size (365.3 ± 57.9 g) were observed in patients with graft loss (*p* < .03, vs. surviving grafts). Significantly higher DSA‐mean fluorescence intensity (MFI) values were present in patients with graft loss (*p* = .0012, vs. surviving grafts).

**Conclusions:**

Patients with preformed DSAs exhibited worse graft outcomes in living donor liver transplantation. Higher DSA‐MFI values and smaller graft size were associated with worse outcomes in LCT‐positive patients. High‐risk patients with preformed DSAs should be considered for appropriate graft selection and application of a desensitization protocol.

## Graphical abstract

This study highlights the clinical impact of preformed donor‐specific antibodies (DSA) in living donor liver transplantation. We interestingly showed that the graft volume is an important risk factor in highly DSA‐mean fluorescence intensity (MFI) cases. An appropriate graft selection should be carefully considered in high‐risk case with preformed DSA in living donor liver transplantation

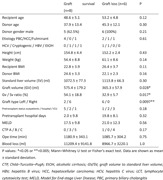

**Abbreviations**
DSA: donor‐specific anti‐HLA antibody
FCXM: flowcytometric crossmatch test
GV/SV: the graft volume to standard liver volume
LCT: lymphocyte cytotoxicity test
MELD: model for end‐stage liver disease
MFI: mean fluorescence intensity
PBC: primary biliary cholangitis

## 1 INTRODUCTION

Preformed donor‐specific anti‐HLA antibodies (DSAs) have a deleterious impact on survival in patients undergoing heart and kidney transplantation,^1^, ^2^, ^3^ so the evaluation of preformed DSAs is critical when selecting a suitable donor.^4^ However, the roles of preformed DSAs in liver transplantation remain controversial.^5^ The liver is a relatively large organ with an unconventional sinusoidal microvascular bed that expresses HLA classes I and II antigens, which presumably absorb allo‐antibodies^6^; the liver also secretes class I HLA antigen, which can facilitate the clearance of DSAs.^7^ Furthermore, the liver has a regenerative capacity and consists of Kupffer cells, which may clear the DSA‐binding soluble class I HLA antigen.^6^ Therefore, the liver may exhibit resistance to antibody‐mediated injury. Furthermore, in terms of first liver transplantation (i.e., excluding re‐transplantation), preformed DSAs do not affect graft survival.^8^ Despite prior reports that preformed DSAs have no impact on liver transplant outcome,^9^, ^10^ previous studies have demonstrated that the presence of DSA positivity in Luminex single‐antigen bead assays is associated with worse outcomes in deceased donor liver transplantation.^11^, ^12^

Thus far, preformed DSAs have been associated with a worse prognosis in patients undergoing deceased donor liver transplantation, compared with patients undergoing living donor liver transplantation.^13^ A previous study using the A2ALL clinical database (2004–2010; 129 living donor liver transplants and 66 deceased donor liver transplants) showed that preformed DSAs were significantly associated with a higher rate of graft loss (*n* = 9 graft losses, *p* < .01; 1‐year graft survival rate: <60%); this association was not observed in patients who underwent living donor liver transplantation. Cold ischemia time is 5–10‐fold shorter in living donor liver transplantation than in deceased donor liver transplantation^13^, ^14^; this finding suggests that longer cold ischemia time may promote antibody‐mediated graft damage. Indeed, the presence of HLA antibodies combined with a longer cold ischemia time has been associated with transplant arteriosclerosis in human vessels.^15^ Thus, shorter cold ischemia time in living donor liver transplantation could theoretically attenuate antibody‐mediated damage. Furthermore, because living donors are usually family members, pregnancy may cause paternal antigen sensitization in which re‐encounter of the same sensitized antigen of graft organ from the spouse or offspring can lead to robust graft rejection.^16^ Additionally, compared with whole‐liver transplants in patients undergoing deceased donor liver transplantation, the smaller graft volume used in living donor liver transplantation is presumably detrimental because whole‐liver transplants contain a sufficient vascular bed that presumably aids in antibody absorption. Herein, we evaluated the role of preformed DSAs in patients undergoing living donor liver transplantation in our institute.

## 2 METHODS

### 2.1 Patients

The study protocol followed the ethical guidelines of the 1975 Declaration of Helsinki and was approved by the institutional review board at Hokkaido University Hospital (#017‐0104). We retrospectively evaluated 175 patients who underwent ABO‐compatible living donor liver transplantation between July 1997 and January 2016, and were followed up in Hokkaido University Hospital. All patients underwent lymphocyte crossmatch tests, including a lymphocyte cytotoxicity test (LCT) and flowcytometric crossmatch test (FCXM), using donor lymphocytes and recipient sera. The cut‐off thresholds of LCT and FCXM were both defined as 20%. We also evaluated LCT‐positive and DSA‐negative recipient sera from stored samples using a single‐antigen bead assay (Labscreen®, One Lambda Inc.). A normalized mean fluorescence intensity (MFI) greater than 1000 was regarded as a positive result in the single‐antigen bead assay. Nine patients were excluded because of death related to the following apparent surgical complications after liver transplantation: hepatic arterial thrombosis (*n* = 2), hepatic aneurysm rupture (*n* = 2), hepatic vein stenosis (*n* = 3), uncontrollable hemorrhage during liver transplantation (*n* = 1), and complications of central venous insertion (*n* = 1). Two patients were excluded because of missing data. After single‐antigen bead assays, DSAs were verified by high‐resolution HLA typing of the donor and recipient, in accordance with a consensus guideline.^17^

### 2.2 HLA typing

Before transplantation, all recipients and donors were typed for HLA‐A, ‐B, ‐Cw, ‐DP, ‐DQ, and ‐DR using LabType SSO® (One Lambda Inc.).

### 2.3 Immunosuppressant protocol

As induction therapy, basiliximab was applied for patients who underwent living donor liver transplantation beginning in October 2003 in Hokkaido University Hospital, in conjunction with triple immunosuppressant therapy that included tacrolimus (from Day 3 after transplantation, target trough 10–15 ng/ml for 1 month after liver transplantation), mycophenolate mofetil (500–2000 mg daily, orally from Day 1), and methylprednisolone (20 mg daily with withdrawal on a weekly basis). Tacrolimus‐based regimens (with or without mycophenolate mofetil or methylprednisolone) were usually applied during the maintenance phase. A desensitization protocol using rituximab was not used among patients eligible for this study.

### 2.4 Statistical analysis

Patient characteristics are represented as medians and interquartile ranges. Continuous variables were evaluated using the Mann–Whitney *U*‐test. The frequencies of categorical variables were evaluated by Fisher's exact test, and the log‐rank test was used to assess graft survival. All statistical analyses were performed using JMP Pro 14. A *p*‐value < .05 was considered statistically significant.

## 3 RESULTS

### 3.1 Incidence of preformed DSAs and characteristics of preformed DSA‐positive patients

**Figure 1:**
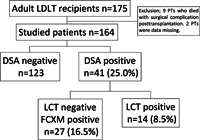
Prevalences of donor‐specific antibodies (DSAs)‐positive findings detected by lymphocyte cytotoxicity test (LCT) and flow cytometric crossmatch (FCXM) assays. Adults who underwent living donor liver transplantation in our institute during the period from 1998 to 2016 (*n* = 175) were retrospectively evaluated. Eleven patients were excluded from this study (nine died of apparent surgical complications and two were missing data concerning preformed DSAs). FCXM‐positive patients were defined as those with DSAs only detected in FCXM examination (i.e., LCT‐negative findings)

Among 164 adult patients who underwent living donor liver transplantation during the study period, 41 (25.0%) had preformed DSAs (Figure 1). Of these DSA‐positive patients, 27 had FCXM‐positive findings alone (16.5%, Figure 1). Fourteen patients (8.5%) had LCT‐positive findings; all also had FCXM‐positive findings. The patient characteristics are shown in Table 1. All 14 patients with pretransplant LCT‐positive findings were women and a significantly greater proportion (42.9%) of these patients exhibited primary biliary cholangitis (PBC, *n* = 6). Among the LCT‐positive patients, 50% of living donors were spouses; this suggested that sensitization might occur following pregnancy. The mean age of LCT‐positive patients was significantly younger (50.5 ± 5.3 years) than the mean age of FCXM‐positive patients (54.1 ± 8.8 years, Table 1). The pretransplant hospital stay tended to be shorter (11.6 ± 14.2 days) in LCT‐positive patients than in FCXM‐positive patients, but no differences in Model for End‐Stage Liver Disease (MELD) and Child–Pugh scores were observed among the groups. Significantly shorter stature, smaller body surface area, and smaller standard liver volume (SV) were observed in DSA‐positive patients, compared with DSA‐negative patients. However, the ratio of graft volume (GV) to SV (i.e., GV/SV ratio) was comparable among the groups. Furthermore, significantly shorter cold ischemia time was observed in LCT‐positive patients.

**Table 1:** Characteristics of preformed DSA‐positive patients who underwent LDLT between July 1997 and January 2016, in Hokkaido University Hospital

		DSA‐negative (*n* = 123)	“DSA‐positive” (*n* = 41)		*p*
			FCXM‐positive (*n* = 27)	LCT‐positive (*n* = 14)	
Sex, female, # (%)	Recipient	49 (39.8)	19 (70.4)	**14 (100.0)	**0.001 versus non‐DSA
	Donor	43 (35.0)	9 (33.3)	3 (21.4)	0.66
Age	Recipient	50.3 ± 11.6	54.1 ± 8.8	*50.5 ± 5.3	*<0.05 versus FCXM+
	Donor	35.8 ± 11.1	36.7 ± 14.0	41.1 ± 13.0	0.74
Liver disease etiology, # (%)					0.23
HCV/HBV/HCC		23(18.7)/31(25.2)/9(7.3)	3(11.1)/3(11.1)/2(7.4)	1(7.1)/1(7.1)/1(7.1)	PBC or not
EtOH/PBC/PSC		7(5.7)/15(12.2)/4(3.3)	3(11.1)/7(25.9)/2(7.4)	1(7.1)/**6(42.9)/0(0)	**0.0025 versus non‐DSA
Fulminant/AIH/cryptogenic		16(13.0)/3(2.4)/2(1.6)	1(3.7)/2(7.4)/0(0)	2(14.3)/0(0)/2(14.3)	
NASH/ADPKD/Others		4(3.3)/3(2.4)/7(5.7)	1(3.7)/2(7.4)/1(3.7)	0(0)/0(0)/0(0)	
Pre‐LT status					
Outpts/Hospital/ICU, # (%)		52(42.3)/50(40.7)/21(17.1)	6(22.2)/17(63.0)/4(14.8)	6(42.9)/4(28.6)/4(28.6)	0.15
Pre‐LT hospital days		19.3 ± 23.8	27.4 ± 31.6	^#^11.6 ± 14.2	^#^0.08 versus FCXM +
MELD		17.5 ± 10.3	18.8 ± 10.1	19.0 ± 10.6	0.91
CTP A/B/C, # (%)		15(12.6)/38(31.9)/67(55.4)	3(11.1)/6(22.2)/18(66.7)	1(7.1)/3(21.4)/9(64.3)	0.59
Height (cm)	Recipient	163.8 ± 10.2	**156.9 ± 8.1	**153.7 ± 3.8	**<0.0015 versus non‐DSA
	Donor	166.7 ± 8.6	164.2 ± 6.7	163.7 ± 5.6	0.87
Weight (kg)	Recipient	61.7 ± 10.8	*57.3 ± 9.1	57.3 ± 11.4	*<0.05 versus non‐DSA
	Donor	63.0 ± 12.3	59.1 ± 10.0	62.9 ± 7.4	1.0
BSA (m^2^)	Recipient	1.67 ± 0.18	**1.56 ± 0.18	**1.54 ± 0.10	**<0.005 versus non‐DSA
BMI	Recipient	23.0 ± 3.3	23.2 ± 3.2	24.3 ± 4.1	1.3
	Donor	22.6 ± 3.4	21.9 ± 3.3	23.6 ± 3.1	0.42
SV (ml)		1179.0 ± 128.1	*1107.2 ± 126.2	*1090.2 ± 73.3	*<0.05 versus non‐DSA
Graft Left/Right, # (%)		75 (61.0)/48 (39.0)	13 (48.2)/14 (51.9)	8 (57.1)/6 (42.9)	0.47
GV, ml		465.1 ± 133.5	472.9 ± 126.7	485.4 ± 173.8	0.95
GV/SV, %		39.4 ± 10.3	42.8 ± 10.9	45.0 ± 18.0	0.82
Relation to donor, # (%)					
Parents/Spouse/Siblings Offspring/Others		8(6.5)/24(19.5)/20(16.3)	0(0)/6(22.2)/2(7.4)	0(0)/7(50.0)/1(7.1)	0.11
		65(52.9)/6(4.9)	17(63.0)/2(7.4)	6(42.9)/0(0)	
Operation time, min		1034.6 ± 237.0	^#^939.2 ± 117.8	1140.1 ± 318.4	^#^0.065 versus non‐DSA
CIT, min		71.4 ± 38.9	94.0 ± 91.6	**50.9 ± 40.9	**<0.005 versus others
WIT, min		49.0 ± 15.9	43.9 ± 10.9	52.4 ± 15.9	0.10
Blood loss in LT, g		8401.8 ± 8790.8	6332.8 ± 4350.9	^#^10248.2 ± 7093.3	^#^0.071 versus FCXM +
Splenectomy, # (%)		38 (30.9)	9 (33.3)	2 (14.3)	0.39

*Note*: Mann–Whitney *U*‐test or Fisher's exact test. Data are shown as mean ± standard deviation, except where indicated otherwise. #<0.1; *<0.05; **<0.005; ***<0.0001.

Abbreviations: ADPKD, autosomal dominant polycystic kidney disease; AIH, autoimmune hepatitis; CIT, cold ischemia time; CTP, Child–Turcotte–Pugh; DSA, donor‐specific antibody; EtOH, alcoholic cirrhosis; FCXM, flowcytometric crossmatch test; GV/SV, graft volume to standard liver volume; HBV, hepatitis B virus; HCC, hepatocellular carcinoma; HCV, hepatitis C virus; LCT, lymphocyte cytotoxicity test; MELD, Model for End‐stage Liver Disease; NASH, nonalcoholic steatohepatitis; LT, liver transplant; PBC, primary biliary cholangitis; PSC, primary sclerosing cholangitis; WIT, warm ischemia time.

### 3.2 Graft survival rates in DSA‐positive patients, particularly LCT‐positive patients, were significantly worse than graft survival rates in DSA‐negative patients

**Figure 2:**
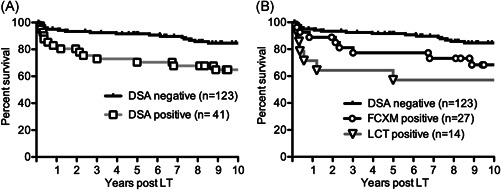
Patient survival of living donor liver transplant recipients with or without preformed donor‐specific antibodies (DSAs). (A) Patients with preformed DSAs (open squares, *n* = 41) had significantly worse patient survival, compared with patients who did not have preformed DSAs (black line, *n* = 123). *p* = .0051, Log‐rank test. (B) Patient survival of patients with preformed DSAs detected by flow cytometric crossmatch (FCXM) alone (open circles, *n* = 27) and lymphocyte cytotoxicity test (LCT) (open triangles, *n* = 14) displayed significantly worse outcomes, compared with DSA‐negative patients (black line, *n* = 123). Log‐rank test. *p* = .0156, FCXM‐positive versus DSA‐negative, *p* = .0357, LCT‐positive versus DSA‐negative

The overall graft survival rate was significantly lower in DSA‐positive patients (*n* = 41) than in DSA‐negative patients (*n* = 123, *p* = .0051, Figure 2A). Among DSA‐positive patients, FCXM‐positive patients (*n* = 27) displayed a worse graft survival rate, compared with DSA‐negative patients (*p* = .0156, Figure 2B). Furthermore, LCT‐positive patients (*n* = 14) exhibited significantly worse graft survival (1‐year survival rate, 71.4%; 5‐year survival rate, 57.1%; *p* = .0357 vs. DSA‐negative patients, Figure 2B). Eventually, of 14 LCT‐positive patients, six experienced graft loss; of these six patients, four lost the graft within 1 year because of clinical events associated with acute rejection.

### 3.3 Smaller graft volume was associated with worse outcome in pretransplant LCT‐positive patients

To identify the prognostic factors in LCT‐positive patients, we compared patients with graft survival (*n* = 8) to patients who had graft loss (*n* = 6, Table 2). Recipient and donor age were slightly older in patients with graft loss. All lost grafts had been taken from male donors. Three of the six patients with graft loss required intensive treatments in the intensive care unit before transplantation. In contrast, five of the eight patients with graft survival were undergoing outpatient treatment before transplantation. Furthermore, patients with graft loss had slightly higher MELD scores, compared with patients who had graft survival. All six patients with graft loss had received a living left hepatic lobe graft, while six of the eight patients with graft survival had received a living right hepatic lobe graft. Consequently, significantly smaller GV and lower GV/SV ratio were observed in patients with graft loss, compared with patients who had graft survival (Table 2). Additionally, we evaluated risk factors for early graft failure. Of 164 adult recipients who underwent living donor liver transplantation, 14 and 18 patients experienced graft loss within 1 and 3 years post‐transplantation, respectively. We found that preformed DSAs, female sex, smaller liver graft size, lower GV/SV ratio, and engraftment of the left hepatic lobe were significantly associated with early graft loss (Table S1). Taken together, the findings indicated that patients with preformed DSAs, particularly LCT‐positive patients, had a significant risk of graft loss when smaller grafts were transplanted.

**Table 2:** Characteristics of LCT‐positive patients with graft survival and those with graft loss

	Graft survival (*n* = 8)	Graft loss (*n* = 6)	*p*
Recipient age	48.6 ± 5.1	53.2 ± 4.8	0.12
Donor age	37.9 ± 13.4	45.3 ± 12.1	0.30
Donor gender male	5 (62.5%)	6 (100%)	0.21
Etiology PBC/HCC/Fulminant	4/0/1	2/1/1	0.61
HCV/Cryptogenic/HBV/EtOH	0/1/1/1	1/1/0/0	
Height (cm)	154.8 ± 4.4	152.2 ± 2.4	0.43
Weight (kg)	54.4 ± 8.8	61.1 ± 8.6	0.14
Recipient BMI	22.8 ± 3.9	26.4 ± 3.7	0.11
Donor BMI	24.6 ± 3.3	22.1 ± 2.3	0.16
Standard liver volume (SV) (ml)	1072.5 ± 77.3	1113.8 ± 66.3	0.30
Graft volume (GV) (ml)	575.4 ± 179.2	365.3 ± 57.9	0.028*
Gv/Sv ratio (%)	54.1 ± 18.8	32.9 ± 5.7	0.017*
Graft type left/right	2/6	6/0	0.0097**
Pretransplant status outpatients/hospital/ICU	5/2/1	1/2/3	0.18
Pretransplant hospital days	2.0 ± 9.8	19.8 ± 8.1	0.32
MELD	17.5 ± 9.8	21.0 ± 12.3	0.56
CTP A/B/C	0/3/5	1/0/4	0.17
Ope time (min)	1180.9 ± 343.1	1085.7 ± 304.2	0.75
Blood loss (ml)	11209.4 ± 9141.8	8966.7 ± 3220.1	1.0

*Note*: Mann–Whitney *U*‐test or Fisher's exact test. Data are shown as mean ± standard deviation. *<0.05; **<0.005.

Abbreviations: CTP, Child–Turcotte–Pugh; EtOH, alcoholic cirrhosis; GV/SV, graft volume to standard liver volume; HBV, hepatitis B virus; HCC, hepatocellular carcinoma; HCV, hepatitis C virus; LCT, lymphocyte cytotoxicity test; MELD, Model for End‐stage Liver Disease; PBC, primary biliary cholangitis.

### 3.4 Higher DSA‐MFI values were associated with worse outcomes in pretransplant DSA‐positive patients

**Figure 3:**
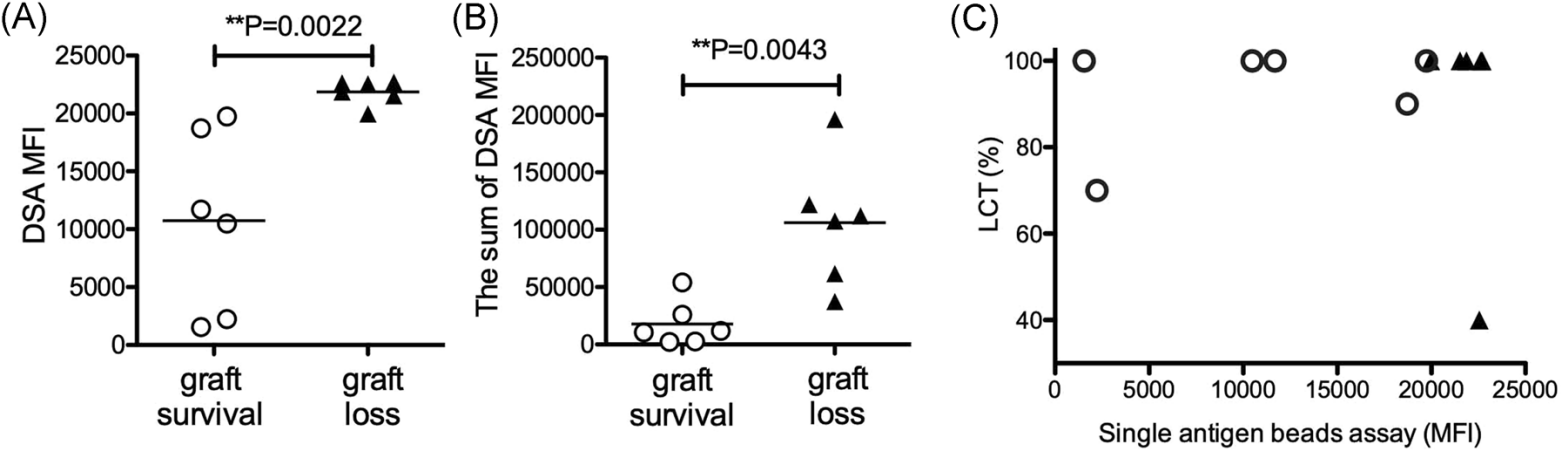
Evaluation of donor‐specific antibody (DSA)‐mean fluorescence intensity (MFI) values in patients with preformed DSAs detected by lymphocyte cytotoxicity test (LCT). The maximum (A) and sum (B) values of preformed DSAs were compared between patients with graft survival (open circles, *n* = 6) and those with graft loss (filled triangles, *n* = 6). The sum of DSA‐MFI means sum of multiple DSA‐MFI. Mann–Whitney *U*‐test. *p* < .05 indicates significant differences. (C) Correlations between proportions of LCT and maximum of DSA‐MFI values in patients with graft survival (open circles, *n* = 6) and those with graft loss (filled triangles, *n* = 6)

**Figure 4:**
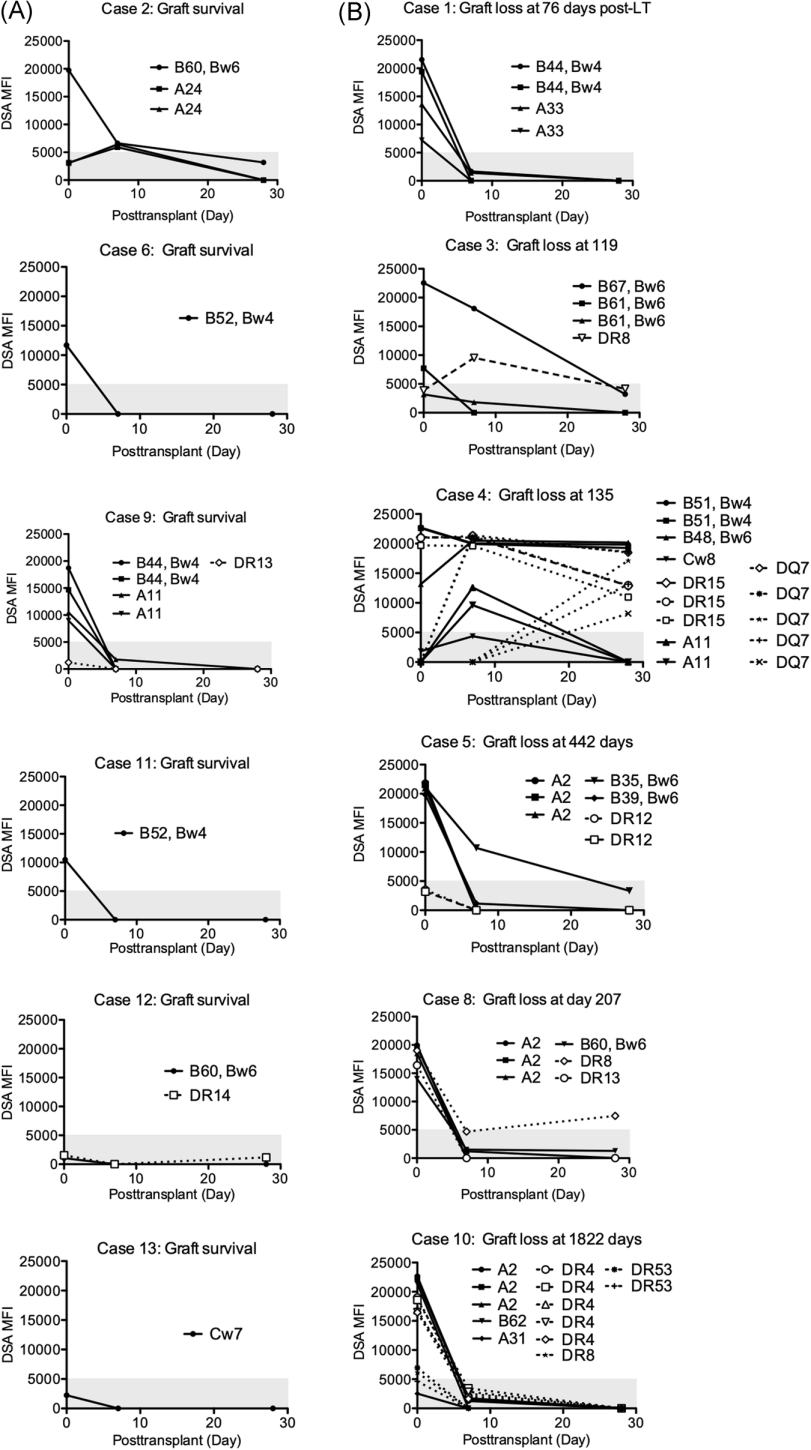
Changes in preformed donor‐specific antibody (DSA)‐mean fluorescence intensity (MFI) values in patients with graft survival (A) and those with graft loss (B) until 1 month after liver transplantation. All data were collected from the patients who exhibited preformed DSAs detected by the lymphocyte cytotoxicity test

We found that DSA‐negative patients displayed a favorable prognosis (Figure 2A). However, a previous study showed that patients with LCT negativity constituted approximately 10% of patients with positive findings in single‐antigen bead assays.^18^ To evaluate DSA negativity using Luminex single‐antigen bead assays, we re‐evaluated stored samples from 67 DSA‐negative patients since 2003. We identified six patients (8.9%) with positive findings in single‐antigen bead assays among DSA‐negative patients who had been evaluated by both FCXM and LCT assays (Table S2). Five of six patients had class I DSAs. Two of six patients had DSA‐MFI values >10,000. Although five of six patients received live donor grafts of the left hepatic lobe, the graft survival rate was favorable among all six patients (5‐year graft survival rate: 100%). This finding suggested that DSA positivity, evaluated by Luminex single‐antigen bead assays in patients with DSA‐negative findings according to LCT and FCXM, does not have a clinical impact with respect to graft survival. Furthermore, we used single‐antigen bead assays to retrospectively assess frozen serum samples collected before liver transplantation from LCT‐positive patients; samples were unavailable for two patients with graft survival. All six patients had experienced graft loss; they exhibited significantly higher maximum pretransplant DSA‐MFI values, compared with values in living patients (Figure 3A). In addition, DSA‐MFI values of two living patients were below 5000, a clinically significant cut‐off value according to the Banff criteria.^19^ Notably, five of six patients with graft loss exhibited both anti‐class I and II DSAs (Figure 4). Furthermore, two of six patients with graft survival had anti‐class II DSAs alone, two had anti‐class I DSAs alone, and two had both anti‐class I and II DSAs. These findings suggested that worse prognosis may be associated with the presence of multiple DSAs. Indeed, a significantly higher DSA‐MFI value was observed in patients with graft loss, compared with patients who had graft survival (Figure 3B). Additionally, the proportion of LCT‐positive patients was not closely associated with the proportion of patients with high DSA‐MFI values (i.e., LCT findings were not strongly linked to single‐antigen bead assay findings). One LCT‐positive patient with graft loss exhibited DSA‐MFI values >20,000 and LCT positivity >40% (filled triangles in Figure 3C). Among patients with graft survival who exhibited LCT positivity 100%, various DSA‐MFI values were evident (open circles in Figure 3C); only two of these patients had DSA‐MFI values near 20,000. Thus, the single‐antigen bead assay demonstrates greater clinical outcome predictability, compared with the LCT.

### 3.5 Clinical course of preformed DSAs until 1 month after liver transplantation exhibited reduction and disappearance in patients with graft survival

Serum samples were obtained from patients with pretransplant DSA positivity on 7 and 28 days after liver transplantation; these samples were assessed using the single‐antigen bead assay. The DSAs in four patients with graft survival had disappeared by 7 days after liver transplantation (Figure 4A). However, no patients exhibited preformed DSA disappearance on Day 7 after liver transplantation among all six patients with graft loss (Figure 4B). DSAs were below the limit of detection at 28 days after liver transplantation in only two of six patients with graft loss (Figure 4B). DSA‐MFI values >5000 at 28 days after liver transplantation were evident in three patients who exhibited graft loss within 1 year after liver transplantation. The DSAs remaining at 28 days included class II antibodies.

## 4 DISCUSSION

In this study, we showed that LCT‐positive patients had poor living donor liver transplantation outcomes. In particular, preformed DSAs with higher MFI values were significantly correlated with worse graft outcomes. Importantly, smaller graft volume was associated with worse prognosis. These data suggest that graft volume is critical for living donor liver transplant recipients with high levels of preformed DSAs.

In patients undergoing liver transplantation, the prevalence of DSA positivity detected by the single‐antigen bead assay is reportedly 7.2%–10.5%.^18^, ^20^ Additionally, a slightly higher proportion (19.6%–33.3%) of pretransplant DSAs have been detected by FCXM, compared with the single‐antigen bead assay,^21^, ^22^ presumably because of sensitivity differences. For patients undergoing living donor liver transplantation in Japan, the vast majority of living donors are family members.^23^ Pregnancy may be a risk factor for sensitization in the context of living donor liver transplantation.^12^ However, the prevalence of preformed DSAs varies in patients undergoing living donor liver transplantation (11.4%–38%).^9^, ^24^ A study using a nationwide multicenter database revealed no significant differences in preformed DSA prevalence between living donor liver transplantation and deceased donor liver transplantation.^13^ Consistent with these findings, our study demonstrated comparable frequencies (25.0% of DSA‐positive patients and 8.5% of LCT‐positive patients), relative to the findings in previous studies.^18^, ^22^ Furthermore, we demonstrated that women with PBC were likely to exhibit LCT positivity. A study performed at another institute in Japan supported our finding that PBC was a risk factor for DSA formation.^24^ However, there are conflicting data concerning the relationship between PBC and sensitization.^25^ There are reportedly no differences in the prevalences of preformed DSAs between patients with PBC and patients with other diseases.^25^, ^26^ Because the exact pathogenesis of PBC remains unclear, further studies of PBC, include its interactions with the immune system, may explain its association with DSA formation in affected patients.

A previous study showed that the presence of complement combined with DSAs is associated with worse prognosis in liver transplant recipients.^11^, ^27^ In our study, six patients with DSA negativity, evaluated by both LCT and FCXM, exhibited DSA‐positive findings according to Luminex single‐antigen bead assays (Table S2). However, these DSA‐positive findings according to Luminex bead assays were not associated with clinical impacts, suggesting that assays to detect complement‐fixing antibodies may be more appropriate for predicting graft survival.^28^ In addition, higher DSA‐MFI values have been associated with worse prognosis^11^, ^28^, ^29^; similarly, we found that higher DSA‐MFI values were significantly associated with graft failure (Figure 3). DSA‐MFI values indicate the extent of antibody binding to antigen‐coated beads, but they do not represent quantitative assessments of antibody levels. Nonetheless, in many instances, the DSA‐MFI value is correlated with the amount of DSAs.^30^ Indeed, a linear correlation has been observed between IgG‐MFI values up to 10,000 and titer values.^30^ Furthermore, higher DSA‐MFI values (e.g., >10,000) have been linked to worse clinical outcomes.^5^, ^12^, ^26^ A high MFI value of preformed class II DSAs has been associated with worse graft survival.^18^, ^31^ Our data demonstrated that preformed class I DSAs generally disappeared after liver transplantation in patients with graft survival, whereas preformed class II DSAs persisted on Day 28 after liver transplantation in patients with graft loss (Figure 4). With recent developments in antibody assessment, future investigations of class II DSA amounts may provide accurate clinical risk prediction.

The underlying mechanisms by which the liver can overcome antibody‐mediated damage include antibody absorption.^32^ Sensitized recipients overcome the presence of DSAs following simultaneous liver kidney transplantation.^6^ In addition, to utilize the antibody “sponge effect” of liver grafts, partial liver transplantation has been performed in sensitized patients for kidney transplantation.^33^ Antibody absorption is presumably promoted in the large capillary beds in the liver, such that the relatively smaller vessel area in partial liver grafts may reduce the capacity for DSA absorption, compared with whole‐liver grafts. A previous study of histological findings at 60–90 min after reperfusion in liver transplantation revealed that crossmatch‐positive patients displayed platelet margination in central veins and sinusoids.^34^ The formation of the DSA‐HLA complex on endothelial cells triggers complement activation and microvasculature destruction.^35^ In addition, there is evidence of C4d deposition shortly after liver transplantation in crossmatch‐positive patients.^36^ DSA binds to HLA expressed on capillary endothelial cells. The peritubular capillary bed in the kidney (0.21 m^2^) is one‐hundredth of the liver capillary bed (21 m^2^). Members of an expert consensus speculated that the liver size may influence the absorption effect.^6^ Additionally, vascular antibody deposition and platelet thrombi in the microvasculature promote tissue ischemic damage. However, Kupffer cell phagocytosis reduces immune complex formation by modifying platelet aggregation.^37^ Resistance to antibody‐mediated injury may depend on the balance between space in vascular beds containing Kupffer cells and the amounts of corresponding antibodies. Indeed, in our study, patients 4 and 5 experience earlier graft loss; both exhibited DSAs at 1 month after liver transplantation (Figure 4B). Furthermore, these patients had received small grafts (330 and 268 g, respectively). Because a small liver graft volume is a critical limitation to overcoming earlier clinical events (e.g., acute rejection and infection), it is unclear whether the graft‐mediated antibody absorption is associated with a specific clinical outcome. Larger liver graft size and younger donor age have been shown to provide a significantly superior survival rate in adult living donor liver transplantation.^38^ In contrast, our findings demonstrated that small graft size was a significant risk factor for graft failure in the presence of preformed DSAs, while donor age was not. Although the precise mechanism underlying liver protection against DSAs remains unknown, our data at least partially support the selection of large grafts in high‐risk living donor liver transplant recipients in the presence of high‐titer preformed DSAs.

To provide a promising treatment protocol for sensitized patients with preformed DSAs, desensitized treatment has been attempted in the field of liver transplantation. A previous case report described a successful desensitized treatment protocol for a patient who had multiple preformed DSAs related to multiple instances of re‐transplantation. In that case, various desensitization treatments (e.g., rituximab, plasmapheresis, splenectomy, intravenous immunoglobulin, and bortezomib) had been applied, which enabled the patient to overcome severe antibody‐mediated rejection after transplantation.^39^ A recent study concerning rituximab‐based desensitization treatment for preformed DSAs in patients undergoing liver transplantation has shown that a high dose (>300 mg/m^2^) of rituximab reduced the incidence of antibody‐mediated rejection^40^; that study demonstrated the safety of a desensitization protocol for DSA‐positive recipients undergoing liver transplantation.^40^ Specific indications and a refined treatment protocol for preformed DSAs in patients undergoing liver transplantation require additional studies.

Our study had some limitations. First, these LCT‐positive patients did not undergo a desensitization treatment protocol that included rituximab. Therefore, it is unclear whether the recent development in desensitization treatment using rituximab^41^ affects the prognosis in high‐risk patients with preformed DSAs. Second, the number of patients was small in this study, so the findings should be confirmed in future multicenter, prospective studies. Third, we evaluated DSAs primarily by using LCT and FCXM methods. Recently, anti‐HLA antibodies have been assessed by using the single‐antigen bead technique in most transplant institutes. Nonetheless, a strong correlation has been reported between LCT and single‐antigen bead results.^18^ Indeed, a higher proportion of LCT‐positive patients with graft loss exhibited high DSA‐MFI values (Figure 4C). Pre‐liver transplant single‐antigen bead assays have recently been added to insurance coverage in Japan, so more precise risk stratification by using single‐antigen‐bead assays may be performed in the future.

We concluded that women with PBC should undergo careful evaluation of preformed DSAs before living donor liver transplantation. Furthermore, patients with preformed DSAs and high DSA‐MFI values may be suitable for graft selection and pretransplant treatment with a desensitization protocol.

## AUTHOR CONTRIBUTIONS

Ryoichi Goto and Makoto Ito performed the research. Ryoichi Goto, Makoto Ito, Norio Kawamura, Masaaki Watanabe, Yoshikazu Ganchiku, Toshiya Kamiyama, Tsuyoshi Shimamura, and Akinobu Taketomi designed the research study. Ryoichi Goto and Makoto Ito analyzed the data. Ryoichi Goto, Tsuyoshi Shimamura, and Akinobu Taketomi wrote the paper.

## Supporting information

Supporting information.

Supporting information.

## Data Availability

The data that support the findings of this study are available on request from the corresponding author. The data are not publicly available due to privacy or ethical restrictions.
