# Supplementary material for: The impact of preformed donor‐specific antibodies in living donor liver transplantation according to graft volume
Source: Immun Inflamm Dis. 2022 Jan 22;10(3):e586. doi: 10.1002/iid3.586 (PMC8926496; doi:10.1002/iid3.586)
Supplement: Supplementary file 1 — Supporting information. [file IID3-10-e586-s001.docx]

Supplemental Table 1

Risk factors associated with early graft failure within 1 and 3 years posttransplantation

| Risk factors | P-value | |
| --- | --- | --- |
|  | Early graft failure within | |
|  | 1 year | 3 years |
| Preformed DSA-positive | 0.019* | 0.0075* |
| Pretransplant LCT-positive | 0.005* | 0.020* |
| Pretransplant FCXM-positive | 0.11 | 0.044* |
| Recipient age | 0.79 | 0.79 |
| Recipient female | 0.025* | 0.013* |
| Recipient weight | 0.083 | 0.10 |
| Recipient BMI | 0.32 | 0.50 |
| Recipient pretransplant status: Outpts, Hospital, ICU | 0.16 | 0.11 |
| MELD | 0.15 | 0.11 |
| CTP score | 0.83 | 0.24 |
| Donor age | 0.26 | 0.13 |
| Donor sex | 0.44 | 0.98 |
| Donor weight | 0.25 | 0.48 |
| Donor BMI | 0.25 | 0.32 |
| Graft volume | 0.0007** | 0.0008** |
| Gv/Sv ratio | 0.007* | 0.0094* |
| Graft type: left hepatic lobe | 0.031* | 0.024* |
| Operation time | 0.15 | 0.22 |
| Blood loss in LT | 0.81 | 0.90 |
| CIT | 0.18 | 0.27 |
| WIT | 0.51 | 0.32 |

P values: *<0.05, or **<0.005; Mann–Whitney U test or Fisher’s exact test.

*CIT, cold ischemia time; CTP, Child–Turcotte–Pugh; DSA, donor-specific antibody; FCXM, flowcytometric crossmatch test; GV/SV, graft volume to standard liver volume; LCT, lymphocyte cytotoxicity test; MELD, Model for End-stage Liver Disease; LT, liver transplant; WIT, warm ischemia time*
