# Supplementary material for: The impact of preformed donor‐specific antibodies in living donor liver transplantation according to graft volume
Source: Immun Inflamm Dis. 2022 Jan 22;10(3):e586. doi: 10.1002/iid3.586 (PMC8926496; doi:10.1002/iid3.586)
Supplement: Supplementary file 2 — Supporting information. [file IID3-10-e586-s002.docx]

Supplemental table 2. DSA positive by re-evaluation of Luminex single-antigen beads assay among DSA negative group.

| Case | Age at LT | Sex | Primary liver disease | DSA-MFI max | Antigen DSA | DSA HLA-Class | Graft type | Gv/Sv (%) | Prognosis  (y post-LT) |
| --- | --- | --- | --- | --- | --- | --- | --- | --- | --- |
| #1 | 53 | Male | HCV | 2909 | A | Class I | Right lobe | 46.2 | Died with pancreatic cancer at 13.8 y post-LT |
| #2 | 50 | Female | HBV | 11277 | B | Class I | Left lobe | 42.1 | Alive (17.2y) |
| #3 | 65 | Female | AIH | 1466 | DR | Class II | Left lobe | 34.9 | Alive (11.8y) |
| #4 | 48 | Female | HCV | 1214 | A | Class I | Left lobe | 44.6 | Alive (11.2y) |
| #5 | 48 | Female | NASH | 9887 | A, B | Class I | Left lobe | 36.4 | Alive (9.4y) |
| #6 | 63 | Female | HCV | 20877 | A | Class I | Left lobe | 33.2 | Alive (8.9y) |

*AIH, autoimmune hepatitis; DSA, donor-specific antibody; Gv/Sv, graft volume to standard liver volume; HBV, hepatitis B virus; HCV, hepatitis C virus; NASH, non-alcoholic steatohepatitis; LT, liver transplant*
